# Prognostic values of interim and post-therapy ^18^F-FDG PET/CT scanning in adult patients with Burkitt’s lymphoma

**DOI:** 10.1186/s40880-015-0057-z

**Published:** 2015-12-02

**Authors:** Wen-Xiao Wei, Jia-Jia Huang, Wen-Yu Li, Xu Zhang, Yi Xia, Wen-Qi Jiang, Wei Fan, Zhi-Ming Li

**Affiliations:** State Key Laboratory of Oncology in South China, Collaborative Innovation Center for Cancer Medicine, Sun Yat-sen University Cancer Center, Guangzhou, 510060 Guangdong P.R. China; Department of Medical Oncology, Sun Yat-sen University Cancer Center, Guangzhou, 510060 Guangdong P.R. China; Lymphoma Division, Cancer Center, Guangdong General Hospital, Guangdong Academy of Medical Sciences, Guangzhou, 510080 Guangdong P.R. China; Department of Nuclear Medicine, Sun Yat-sen University Cancer Center, Guangzhou, 510060 Guangdong P.R. China

**Keywords:** Burkitt’s lymphoma, Mid-therapy PET/CT, Post-therapy PET/CT, Prognosis, Adult patients

## Abstract

**Background:**

The prognostic values of interim and post-therapy fluorine-18-fluorodeoxyglucose (^18^F-FDG) positron emission tomography (PET) and PET/computed tomography (CT) scanning have been confirmed in several subtypes of lymphoma. However, its prognostic value in Burkitt’s lymphoma has not been clearly defined. The aim of the present study was to assess the prognostic value of PET/CT scanning during different treatment processes of Burkitt’s lymphoma.

**Methods:**

A total of 29 adult patients with newly diagnosed Burkitt’s lymphoma were retrospectively involved in this study; of them, 23 patients underwent baseline PET/CT, 15 patients underwent mid-therapy PET/CT after 1–4 cycles of chemotherapy, and 17 patients underwent post-therapy PET/CT after all planned first-line chemotherapy cycles. Mid-therapy and post-therapy PET/CT results (positive vs. negative) were visually interpreted according to the criteria of the International Harmonization Project. The reduction in the maximum standardizes uptake values (∆SUVmax) of 25%, 50%, and 75% were regarded as cutoff points. Overall survival (OS) and progression-free survival (PFS) were regarded as the major endpoints.

**Results:**

The median OS and PFS were 27.6 months (range 6.5–78.3 months) and 27.2 months (range 3.0–78.3 months), respectively. The median SUVmax of the baseline PET/CT was 18.3 (range 1.6–35.9), whereas the median SUVmax of the mid-therapy and post-therapy PET/CT decreased to 4.0 (range 0–17.6) and 3.0 (range 0–14.5), respectively. The patients’ Eastern Cooperative Oncology Group (ECOG) scores (<2 vs. ≥2) were significantly associated with the baseline PET/CT SUVmax. The mid-therapy and post-therapy PET/CT results (positive vs. negative) showed no significant association with OS or PFS. The optimal cutoff ∆SUVmax from the baseline to the post-therapy PET/CT that could predict a change in OS in patients with Burkitt’s lymphoma was 50% (*P* = 0.019).

**Conclusions:**

^18^F-FDG uptake was intense in Burkitt’s lymphoma, and there was a significant reduction in SUVmax during the interim and post-therapy PET/CT procedures. A ∆SUVmax of greater than 50% was a favorable cutoff point to predict the OS of Burkitt’s lymphoma patients.

## Background

Burkitt’s lymphoma is a rare and highly aggressive type of B-cell non-Hodgkin’s lymphoma (NHL). It is divided into endemic, sporadic, and immunodeficiency-associated variants [1–3] and has an intimate relationship with Epstein-Barr virus infection [4, 5]. Burkitt’s lymphoma accounts for 40% of pediatric lymphomas and less than 5% of adult lymphoma cases [6]. Highly aggressive neoplasms are potentially curable with short and intensive treatment schedules, with survival rates of up to 90% [2, 7, 8].

Fluorine-18-fluorodeoxyglucose (^18^F-FDG) positron emission tomography (PET) and PET/computed tomography (CT) are used to stage and monitor the response of most lymphomas to therapy during clinical treatment, and their use has become increasingly prevalent [9–11]. Recent studies have demonstrated the prognostic value of interim PET or PET/CT performed after 1–4 cycles of chemotherapy for some subtypes of lymphoma. A positive early interim ^18^F-FDG PET was shown to identify poor responders in patients suffering from advanced-stage or extranodal disease [12]. In a prospective study of patients with high-risk diffuse large B-cell lymphoma (DLBCL), the reduction in the maximum standardized uptake values (∆SUVmax) between the baseline measurement and the measurements after 2 and 4 cycles of treatment was feasible for high-risk DLBCL and more accurately predicted the patient’s outcome than visual analysis [13]. In a recent report regarding patients with mature T-cell and natural killer (NK) cell lymphomas, both interim and post-therapy PET/CT SUVmax were independent prognostic predictors [14]. However, studies of the prognostic value of PET or PET/CT in patients with Burkitt’s lymphoma have been limited [15–17]. The purpose of this study was to explore the prognostic value of interim and post-therapy PET/CT in adult patients with Burkitt’s lymphoma.

## Patients and methods

### Patient selection and patient characteristics

Consecutive patients aged 16 and older with newly diagnosed Burkitt’s lymphoma between September 2006 and October 2012 at the Sun Yat-sen University Cancer Center, China were included in this study. Positive human immunodeficiency virus (HIV) status and pregnancy were exclusive criteria. A subset of patients underwent baseline whole-body PET/CT, mid-therapy whole-body PET/CT after 1–4 cycles of chemotherapy, and post-therapy whole-body PET/CT at the end of first-line treatment; all patients underwent at least one of these examinations. Before initiating treatment, the data of demographic analysis, symptoms, presence of bulky disease (tumor diameter >10 cm), B symptoms, a physical examination, Eastern Cooperative Oncology Group (ECOG) performance status, and laboratory tests were collected and a complete assessment was performed. The International Prognostic Index (IPI) score was determined for all patients based on age, ECOG performance status, serum lactate dehydrogenase (LDH) level, Ann Arbor stage, and the number of extranodal sites [18]. Baseline, mid-therapy, and post-therapy PET/CT results were assessed according to the revised International Workshop Criteria [19]. This study protocol was approved by the ethics committees of Sun Yat-sen University Cancer Center. All patients provided informed consent to allow the use of their medical records for research purposes.

## ^18^F-FDG PET/CT scan protocol

Whole-body scans were performed on a combined PET/CT system (Discovery ST, with a 16-slice CT component; GE Healthcare Bio-Sciences Corp, Piscataway, NJ, USA). All patients were instructed to fast for at least 6 h before the administration of 5.55 MBq/kg ^18^F-FDG. Whole-body acquisition began 45–60 min after ^18^F-FDG injection, and the patient was scanned from the groin up to the head at the mid-thigh level with a 5-min acquisition per bed position. The CT acquisition was obtained at 140 kV and 150–160 mA with a 5-mm section thickness. An Entegra or Xeleris (GE Healthcare, NJ, USA) workstation was used for the registration and fusion of the acquired images from the PET and CT scans. Corrections were applied for random effects, geometry, attenuation, and scatter.

### Image analysis

The original PET images were analyzed by two nuclear medicine physicians who were unaware of the clinical history of the patients. A positive PET/CT scan was defined as a ^18^F-FDG uptake greater than the background activity in the surrounding tissues, which was unrelated to physiological uptake; a negative PET/CT scan was defined as no residual abnormal uptake at any site [19]. The intensity of ^18^F-FDG uptake was assessed on the basis of the standardized uptake value (SUV). The SUVmax was recorded as a statistical criterion to minimize partial volume effects and to ensure the reproducibility of the measurements. The ∆SUVmax was calculated using the following equations: (SUVmax [baseline PET/CT] − SUVmax [mid-therapy PET/CT] or SUVmax [post-therapy PET/CT])/SUVmax [baseline PET/CT]. To determine the percentage of SUVmax reduction, ∆SUVmax was divided by the SUVmax of the baseline PET/CT.

### Statistical methods

Treatment response was evaluated according to the International Working Group Recommendations for Response Criteria for non-Hodgkin’s lymphomas [18]. Overall survival (OS) was calculated from the date of diagnosis to the date of death from any cause or to the date of final follow-up. Progression-free survival (PFS) was calculated from the date of diagnosis to the date of first disease progression, relapse after response, death from any cause, or final contact. ∆SUVmax of 25%, 50%, and 75% were regarded as cutoff points. The associations of PET/CT results with prognostic factors were analyzed by using a Mann–Whitney *U* test. Survival curves were plotted according to the Kaplan–Meier method, and differences between groups were analyzed by using a two-tailed log-rank test. A *P* value of <0.05 was considered statistically significant. All statistical analyses were performed using SPSS software for Windows, version 19.0 (SPSS Inc., Chicago, IL, USA).

## Results

### Patient characteristics and outcome

A total of 29 patients were included and assessed in this study. The age of onset ranged from 16 to 71 years, with a median age of 39 years. The male to female ratio was 1.9 (19 males and 10 females). The main clinical features of the 29 patients are detailed in Table 1. All patients were histologically diagnosed with Burkitt’s lymphoma, including 2 patients with Burkitt’s-like variants. All patients received 2–8 cycles (median, 6 cycles) of first-line chemotherapy. Twenty-four patients underwent intensive chemotherapy, such as CODOXM/IVAC (cyclophosphamide, vincristine, doxorubicin, methotrexate, ifosfamide, etoposide, and cytarabine) and HyperCAVD (hyperfractionated cyclophosphamide, vincristine, doxorubicin, and dexamethasone), and 5 patients received moderate-intensity protocols such as CHOP (doxorubicin, vincristine, cyclophosphamide, and prednisone). A total of 21 patients (72.4%) received both chemotherapy and rituximab. In total, 22 patients achieved complete response (CR), 4 achieved partial response (PR), 2 died, and 1 showed progressive disease (PD) but still survived up to the last follow-up. The median OS was 27.6 months (range 6.5–78.3 months), and the median PFS was 27.2 months (range 3.0–78.3 months).

**Table 1:** Patient characteristics according to the PET/CT SUVmax at different treatment time points

Variable	Baseline SUVmax		*P* value	Mid-therapy SUVmax		*P* value	Post-therapy SUVmax		*P* value
	n	Mean ± SD		n	Mean ± SD		n	Mean ± SD	
Age			0.073			0.489			0.695
<60 years	20	18.5 ± 8.5		13	4.1 ± 4.9		14	3.2 ± 3.9	
≥60 years	3	7.1 ± 2.6		2	6.3 ± 7.4		3	2.7 ± 4.6	
Sex			0.357			0.595			0.411
Male	17	17.5 ± 8.5		11	4.0 ± 3.6		13	3.0 ± 2.6	
Female	6	15.5 ± 10.3		4	5.3 ± 8.4		4	3.6 ± 7.3	
B symptoms			1.000			0.953			1.000
Yes	8	17.7 ± 9.1		7	3.3 ± 3.0		8	2.7 ± 2.5	
No	15	16.6 ± 9.0		8	5.3 ± 6.3		9	3.5 ± 5.0	
Bulky disease^a^			0.069			0.293			0.164
Yes	4	24.1 ± 5.6		3	1.8 ± 3.1		2	4.7 ± 0.6	
No	19	15.5 ± 8.8		12	5.0 ± 5.2		15	2.9 ± 4.1	
ECOG scores			0.014			0.631			0.363
<2	14	13.4 ± 7.6		9	5.2 ± 5.8		10	3.0 ± 4.8	
≥2	9	22.6 ± 8.0		6	3.1 ± 3.4		7	3.3 ± 2.4	
Ann arbor stage			0.068			0.674			0.111
I–IIE	10	14.0 ± 10.5		6	4.6 ± 4.3		9	1.8 ± 2.8	
III–IVE	13	19.4 ± 6.8		9	4.2 ± 5.6		8	4.7 ± 4.6	
LDH			0.156			0.262			0.618
≤245	12	14.9 ± 11.3		7	6.2 ± 6.3		8	3.5 ± 5.3	
>245	11	19.3 ± 4.5		8	2.7 ± 3.0		9	3.0 ± 2.4	
IPI scores			0.114			0.368			0.176
≤2	14	15.0 ± 9.9		9	5.5 ± 5.8		11	2.8 ± 4.6	
>2	9	20.2 ± 6.1		6	2.7 ± 3.1		6	3.8 ± 2.2	

*PET* positron emission tomography, *CT* computed tomography, *SUVmax* the maximum standardized uptake value, *ECOG* eastern cooperative oncology group, *LDH* lactate dehydrogenase, *IPI* international prognostic index, *SD* standard deviation

^a^Bulky disease, tumor diameter >10 cm

### Survival analysis of SUVmax

A total of 23 patients were exposed to baseline PET/CT and demonstrated intense uptake foci, with a median SUVmax of 18.3 (range 1.6–35.9). In total, 15 patients underwent mid-therapy PET/CT after 1–4 cycles of chemotherapy (median, 3 cycles) and 17 underwent post-therapy PET/CT after all planned first-line chemotherapy, with a median SUVmax of 4.0 (range 0–17.6) and 3.0 (range 0–14.5), respectively (Table 2).

**Table 2:** SUVmax at different treatment time points of PET/CT

PET/CT	No. of patients	SUVmax		
		Mean ± SD	Range	Median
Baseline	23	17.0 ± 8.8	1.6–35.9	18.3
Mid-therapy	15	4.4 ± 5.0	0–17.6	4.0
Post-therapy	17	3.1 ± 3.9	0–14.5	3.0

*PET* positron emission tomography, *CT* computed tomography, *SUVmax* the maximum standardized uptake value, *ECOG* eastern cooperative oncology group, *LDH* lactate dehydrogenase, *IPI* international prognostic index, *SD* standard deviation

To explore whether some prognostic factors were associated with various SUVmax at each time point, several parameters, including age, sex, B symptoms, bulky disease, ECOG score, Ann Arbor stage, LDH level, and IPI score, were analyzed by using a Mann–Whitney *U* test. The analysis revealed significant differences in the baseline PET/CT SUVmax between different ECOG score groups (<2 vs. ≥2, *P* = 0.014), but not for other groups of prognostic factors. No significant differences in the mid-therapy or post-therapy PET/CT SUVmax were observed for any groups of prognostic factors (Table 1).

A total of 10 patients had positive mid-therapy PET/CT results, and 5 patients had negative mid-therapy PET/CT results. The mid-therapy PET/CT results had no influence on OS and PFS (*P* = 0.083 and *P* = 0.317, respectively). The median OS and PFS appeared to be longer in patients with negative post-therapy PET/CT results than in those with positive post-therapy PET/CT results, but this difference was not statistically significant (*P* = 0.403 and *P* = 0.777, respectively).

### Survival analysis of ∆SUVmax

Based on the mid-therapy and post-therapy PET/CT results, we defined ∆SUVmax of 25%, 50%, and 75% as cutoff points. There were no significant differences in OS or PFS between the groups of the ∆SUVmax greater than 25%, 50%, and 75% and those of the ∆SUVmax no greater than 25%, 50%, and 75%, respectively, from the baseline PET/CT to mid-therapy PET/CT (for OS, *P* = 0.083, 0.083, and 0.317, respectively; for PFS, *P* = 0.351, 0.351, and 0.116, respectively). For ∆SUVmax from the baseline PET/CT to post-therapy PET/CT, the median OS of patients with the ∆SUVmax greater than 50% was longer than that of patients with the ∆SUVmax no greater than 50% (42.4 vs. 6.5 months, *P* = 0.019). A post-therapy PET/CT ∆SUVmax of 50% was an optimal cutoff for predicting OS, whereas a post-therapy PET/CT ∆SUVmax of 25% or 75% was unable to predict OS (*P* = 0.773 and *P* = 0.068, respectively). The ∆SUVmax tended to have greater accuracy in predicting PFS, whereas the ∆SUVmax of greater than 50% and that of 50% or less had no significant difference in statistics (the median PFS, 34.8 vs. 5.9 months, *P* = 0.349). The post-therapy PET/CT ∆SUVmax of 25% or 75% was unable to predict PFS (*P* = 0.584 and *P* = 0.685, respectively). Univariate regression analysis results indicated that only the cutoff point of 50% ∆SUVmax from the baseline PET/CT to post-therapy PET/CT had statistical significance for OS (*P* = 0.019). However, multivariate COX regression analysis showed that none of these facts had a significant impact on OS or PFS.

## Discussion

Burkitt’s lymphoma is usually indicated by intense ^18^F-FDG uptake in untreated lymphomatous lesions due to a high glycolytic rate. Accurate initial staging plays an important role in the selection and duration of treatment. The roles of ^18^F-FDG PET-PET/CT in staging, treatment response evaluation, and prognostic prediction for many types of lymphomas have been explored [12–14]. However, there are very limited data available on the role and prognostic value of interim and post-therapy ^18^F-FDG PET/CT in adult Burkitt’s lymphoma patients.

Almost all studies of PET/CT in Burkitt’s lymphomas have involved individual cases. The prognostic value of mid-therapy and post-therapy PET/CT has rarely been reported. Barrington et al. [20] reported that a 32-year-old Burkitt’s lymphoma patient presented with intense ^18^F-FDG uptake in the bone marrow, bowel, and peritoneum on PET-only scan. A repeat PET scan was negative 6 days after the commencement of chemotherapy [20]. Chander et al. [21] reported a 34-year-old male patient with PET-positive head and neck Burkitt’s lymphoma who underwent a slight decrease in SUVmax at the post-therapy follow-up PET, suggesting a PR to the chemotherapy. Ford et al. [22] demonstrated a false-positive restaging PET scan in the spleen of a 58-year-old man with Burkitt’s lymphoma. Wang et al. [23] reported a 7-year-old boy with ^18^F-FDG-avid lesions in the pleura that were identified by PET/CT, lesions in the peritoneum, mesentery, and omentum with marked ascites, and mesenteric nodules and renal involvement as demonstrated by CT.

In the present study, 23 patients received baseline PET/CT examinations, and these patients were highly ^18^F-FDG-avid at initial presentation, with a median SUVmax of 18.3. The highly aggressive nature of Burkitt’s lymphoma leads to intense ^18^F-FDG uptake during the initial stage. These results are consistent with those of Schöder et al. [24], who suggested that the intensity of ^18^F-FDG uptake was generally higher in aggressive lymphoma than in indolent lymphoma. After 2–8 cycles of chemotherapy with the main CODOXM/IVAC regimen, more than 90% of patients achieved a good response, reflecting the chemosensitivity of the disease. ^18^F-FDG uptake was decreased or even negative during remission after chemotherapy. Karantanis et al. [25] reported that ^18^F-FDG PET examinations in most Burkitt’s lymphoma patients were negative during or immediately after therapy, which is consistent with our findings. Many previous studies have revealed the value of mid-therapy and post-therapy PET/CT in predicting disease progression. In a retrospective study of 85 patients, Hutchings et al. [26] showed that ^18^F-FDG PET after 2–3 cycles of chemotherapy had a high negative predictive value in the early-stage diseases and a high positive predictive value in the advanced-stage disease, independent of other known prognostic factors. Hutchings et al. [12] demonstrated that early interim ^18^F-FDG PET was a stronger predictor of patient outcome than the known prognostic factors and that a positive early interim ^18^F-FDG PET was highly predictive of progression in patients with advanced-stage or extranodal disease. A study of 85 patients with high-risk DLBCL revealed that the outcomes did not significantly differ for PET2 and PET4 scans that were visually positive or negative [13]. According to our data on the relationship between PET/CT and survival, patients with positive post-therapy PET/CT results tended to have poorer outcomes than patients with negative post-therapy PET/CT results, but this difference was not significant. However, these studies had limited sample sizes, and more definitive answers to this question could be obtained through large-scale studies.

We investigated the relationships between PET/CT results and prognostic factors. The results showed different ECOG scores in patients were significantly associated with the baseline PET/CT SUVmax, which is consistent with the results by Chihara et al. [27].

Various factors are related to the scan quality. Effective control should be mandatory, and the injection-to-scanning time should be fixed. To a lesser extent, SUVmax analysis can generate false-positive results because ^18^F-FDG uptake is not only specific for tumor cells but also observed in inflammatory and infectious processes or after bone marrow stimulation. Quantitative assessment is likely a more objective and effective way to interpret PET/CT results. Numerous previous studies have emphasized the percentage of SUVmax reduction. Itti et al. [28] proposed that an optimal cutoff of 72.9% SUVmax reduction from the baseline to the end of therapy yielded a higher estimated 2-year event-free survival rate in patients with a reduction of more than 72.9% (79% vs. 32%). Casasnovas et al. [13] and Lin et al. [29] regarded ∆SUVmax (between the baseline PET and mid-therapy PET) analysis as a feasibly better predictor of outcome than visual analysis for high-risk DLBCL. Previous studies have established several cutoff points for predicting survival using receiver-operating characteristic analysis. It was found that a ∆SUVmax ≤91.8% was the best cutoff point [30], whereas a ∆SUVmax of >70% prompted favorable prognosis [31]. Combined with previous studies, we defined ∆SUVmax of 25%, 50%, and 75% as cutoff points. In the ∆SUVmax analysis, a 50% ∆SUVmax from the baseline to the end of therapy was an optimal cutoff point. ∆SUVmax of greater than 50% led to a favorable predictive value for OS compared with that of 50% or less. The OS tended to be longer in patients with ∆SUVmax >75% than in those with ∆SUVmax ≤75% although the difference was not significant. Due to the limited survey samples, a long-term and further study should be conducted to help us understand the subject comprehensively and objectively.

In conclusion, our results indicated that ^18^F-FDG uptake was intense in Burkitt’s lymphoma, and the ∆SUVmax was significantly different between the interim and post treatment. The patient’s ECOG score was significantly associated with the patient’s baseline PET/CT SUVmax. A post-therapy PET/CT ∆SUVmax of 50% was an optimal cutoff for predicting OS. Longer follow-up time and an analysis of a larger cohort are warranted to confirm the prognostic value of mid-therapy and post-therapy PET/CT in Burkitt’s lymphoma patients. Furthermore, further work should be perform to unify the response criteria and further research is needed to assess the prognostic value.
